# A Modified Fiberoptic Endoscopic Evaluation of Swallowing Evaluating Esophageal Dysphagia by a Capsule: A Pilot Study

**DOI:** 10.1007/s00455-024-10724-z

**Published:** 2024-06-13

**Authors:** Youval Slovik, Benyamin Meir Kaminer, Gorali Revital, Alona Ron, Mai Harris, Oren Ziv, Ayelet Loutati, Oded Cohen

**Affiliations:** 1 Department of Otolaryngology-Head & Neck Surgery, Soroka University Medical Center, POB 151, Beer-Sheva, Israel; 2 Faculty of Health Science, Ben Gurion University of the Negev, Beer Sheva, Israel; 3 Hearing & Speech Institute, Soroka University Medical Center, Beer-Sheva, Israel; 4 Department of Rehabilation, Soroka University Medical Center, Beer-Sheva, Israel; 5 Department of Brain and Cognitive Sciences, Ben Gurion University of the Negev, Beer-Sheva, Israel; 6 Medical School for International Health, Ben-Gurion University of the Negev, Be’er Sheva, Israel; 7 Department of Otolaryngology-Head and Neck Surgery, Samson Assuta Ashdod University Hospital, Ashdod, Israel

**Keywords:** Dysphagia, Capsule, Fees, Laryngoscope, Swallowing

## Abstract

While functional endoscopic evaluation of swallowing (FEES) is the most useful diagnostic test for the evaluation of dysphagia, it cannot evaluate the esophageal phase of swallowing. To evaluate if a modification for the FEES exam by swallowing an empty capsule and screening of the upper esophagus could be used for early detection of esophageal dysphagia. A prospective, single-center, pilot study. At the end of a standard FEES exam, the patients were asked to swallow an empty capsule. Fifteen seconds later, the endoscope was inserted into the upper esophagus. A pathological capsule test was defined when the capsule was seen in the esophagus. In such cases, the patient was advised to undergo a gastroscopy, MBS, or esophageal manometry, which were compared to the results of the capsule test. The capsule test was utilized in 109 patients. A pathological capsule test was found in 55 patients (57.8%). In 48 patients (87.3%), an isolated or combined esophageal dysphagia was seen. The accuracy value of the capsule test compared to gastroenterology tests was 83.3%, sensitivity 88.46%, specificity 75%, PPV 85%, and NPV 80%. A modification of the standard FEES exam by including an empty capsule swallow test with an upper esophagus examination may provide a useful screening tool for esophageal dysphagia.

## Introduction

Dysphagia is a commonly reported symptom. Approximately 1 million new cases are diagnosed annually in the United States [1, 2] or 1 out of 25 adults, however, only a minority seek care [3]. The prevalence of dysphagia is dependent on age, cause, and the method of diagnosis and is estimated to be 20% in the general population, occurring more frequently in women and older populations [3, 4].

Nowadays, Fiberoptic endoscopic evaluation of swallowing (FEES) is considered the standard approach for oropharyngeal swallowing evaluations in Europe [5, 6]. However, an integral limitation of the FEES exam is its restricted ability to evaluate the esophageal phase of swallowing. The commonly used diagnostic tools for the esophageal phase of swallowing include a Modified barium scan (MBS), esophagoscopy, and esophageal manometry. However, these tests require specific equipment used by a well-trained medical staff and may include exposure to radiation. Although In office esophagoscopy has been gaining popularity in recent years [6, 7], it is relatively expensive and is not as accessible as the FEES exam. Therefore, a modification of the FESS exam, which would examine esophageal dysphagia, may offer an alternative method for the initial assessment of esophageal dysphagia. Such modification may reduce the current diagnostic delay of esophageal dysphagia seen especially among the elderly population [8, 9].

In this pilot study, we aim to evaluate the possibility of diagnosing swallowing disorders related to the esophageal phase of swallowing with a modified FEES exam, using an empty capsule combined with an evaluation of the upper esophagus using a regular flexible laryngoscope.

## Materials and Methods

The study was approved by the Institutional Review Board (trial number 0066-20), and written informed consent was obtained from all participants. This prospective study was conducted at the dedicated dysphagia outpatient clinic between January 1, 2021, and February 30, 2023. The exclusion criteria include patients younger than 18 years, pregnant patients, and patients with intellectual disability.

Our dysphagia outpatient clinic is a joint clinic, including a dedicated laryngologist (Y.S.) and a speech and language pathologist (SLP, A.R.). All participants underwent a standardized otorhinolaryngological examination and a modified FEES exam using ENF-VH HD rhino-laryngovideoscope with the CLL-S1 LED Light source (Olympus, Germany). The diameter of the endoscope was 3.2 mm.

### Routine FEES Examination

Each participant was seated on an examination chair, and the endoscopic examination was recorded. First, a lubricant gel was dripped into the patient’s most patent nare. A flexible endoscope was then inserted through the lubricated nostril and positioned at the oropharynx to allow a comprehensive assessment of the swallowing process. Each participant was examined by ingesting at least three types of food consistencies according to the International Dysphagia Diet Standardization Initiative (IDDSI) scale (Table 1). The food was dyed with a blue food color to optimize the view. The liquids were given to the patients with a teaspoon (5 ml) or as single sips from a glass (according to the patient’s abilities). The semiliquid foods were given with a teaspoon. The size of the solid foods was determined by the patient. The patient was given a slice of the solid food and was asked to take a normal bite. In equivocal cases, or when an evaluation of the efficiency of a compensated maneuver was indicated, the patient was asked to take another dose of the same consistency sample.

**Table 1:** Food consistencies in FEES exam

IDDSI scale	Discreption	The food we used
0	Thin	Milk
1	Slightly thick	NUTREN 2.0 Nestle health science Co
2	Mildly thick	Water with 2 spoons of food thickener
3	Moderately thick	Yogurt
4	Pureed	Applesauce
5	Minced and moist	Cottage cheese
6	Soft and bite-sized	Minced Tuna fish
7 EC	Easy to chew	Gouda cheese
7	Regular	Bread

**Fig. 1:**
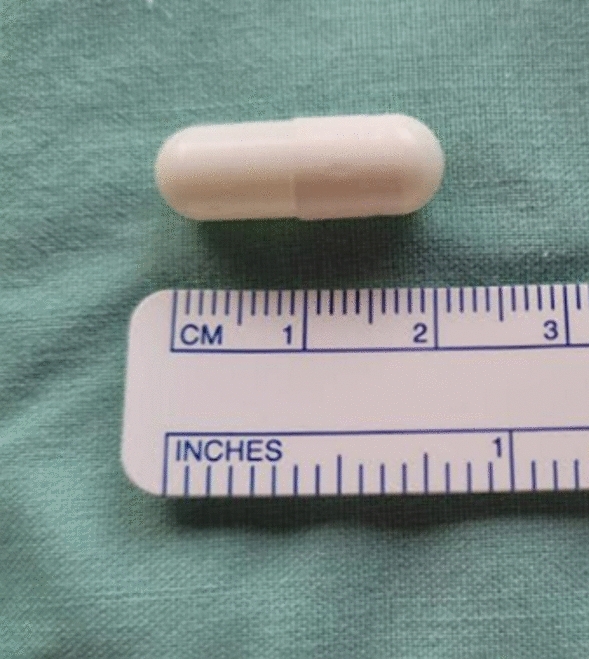
The examined capsule

A modified FEES with an ‘empty capsule’ swallow test was conducted as well. All patients who were appointed for the FEES exam at our clinic were asked to participate in the current pilot study prior to starting their FEES exam. The consent included swallowing an empty capsule made of gelatin, which is followed by an inspection of the upper esophagus while looking for findings suggestive of esophageal dysphagia. The diameter of the capsule was 20 mm and had a volume of 0.6 ml, as shown in Fig. 1.

Fifteen seconds after the capsule was swallowed, an endoscope was inserted 10–15 cm into the esophagus.

Any presence of the capsule in the oral cavity, vallecula, or esophagus was documented as pathological and was categorized as oral, pharyngeal, or esophageal, respectively, or combined (if the capsule was visualized in more than one site).

### Data Collection

Additional information collected included sex, age, and comorbidities. The latter was defined as any chronic or previous disease requiring continuous medical therapy. Comorbidities were grouped as none, 1–3 comorbidities, and > 3 comorbidities. Neurological comorbidities were collected separately and divided into (1) cerebrovascular accident (CVA) history, (2) Parkinson’s disease, and (3) other neurological diseases. Vocal cord status (paresis vs. none) was collected based on fiberoptic examination during FEES. Additional esophageal dysphagia inquiries, including MBS, esophagoscopy, and esophageal manometry, were obtained from the patient’s report. Any pathological findings on these tests were collected as well.

### Statistical Analysis

Continuous variables were expressed as mean ± standard deviation if normally distributed or median with interquartile range if skewed. Categorical variables were presented as frequency (%). Continuous data was compared with the student’s t-test and Mann–Whitney test to compare normally and non-normally distributed continuous variables, respectively. Categorical data was compared using the chi-square test or Fisher exact test. A series of binary classification evaluation tests were conducted to assess the predictive performance of the capsule study. In each iteration, a distinct standard test was employed as the gold standard for establishing accurate predictions. Additionally, the model's performance was assessed through the examination of diverse prediction metrics, including sensitivity, specificity, positive predictive value (PPV), negative predictive value (NPV), and overall accuracy. All statistical analyses were performed using R software version 3.4.4 (R Foundation for Statistical Computing). An association was considered statistically significant for a two-sided P value of less than 0.05.

## Results

A total of 154 patients underwent FEES in the study period. The distribution of patients’ age, gender, and comorbidities are shown in Table 2. The average age of participants was 65.9 ± 17.03 years and included 93 males (60.38%). The majority of the cohort (88 patients, 57.1%) had 1–3 comorbidities. A total of 64 patients (41.5%) were diagnosed with neurological comorbidities.

**Table 2:** Patients’ characteristics

	Total (n = 109)	Capsule positive (n = 54)	Capsule negative (n = 55)	p Value
Age (years, mean ± SD)	66.9 ± 16.8	70.5 ± 12.1	63.3 ± 19.9	0.116
Gender				0.132
Female	41(37.6%)	25 (45.5%)	16 (29.6%)	
Male	68(62.4%)	30(54.5%)	38 (70.4%5)	
Comorbidities				< 0.001
0	26 (23.9%)	20 (37.0%)	6 (10.9&)	
1–3	66 (60.6%)	23 (42.6%)	43 (78.2%)	
> 3	17 (15.6%)	11 (20.4%)	6 (10.9%)	
DM				0.0298
Negative	73 (67.0%)	31 (56.4%)	42 (77.8%)	
Positive	36 (33.0%)	24 (43.6%)	12 (22.2%)	
Neuro				0.652
CVA	24 (22.0%)	14 (25.5%)	10 (18.5%)	
PD	7 (6.4%)	4 (7.3%)	3 (5.6%)	
Other	11 (10.1%)	4 (7.3%)	7 (13.0%)	
None	67 (61.5%)	33 (60.0%)	34 (63%)	
Vocal cord paresis				1
Negative	103 (94.5%)	52 (94.5%)	51(94.4%)	
Positive	6 (5.5%)	3 (5.5%)	3 (5.6%)	

**Fig. 2:**
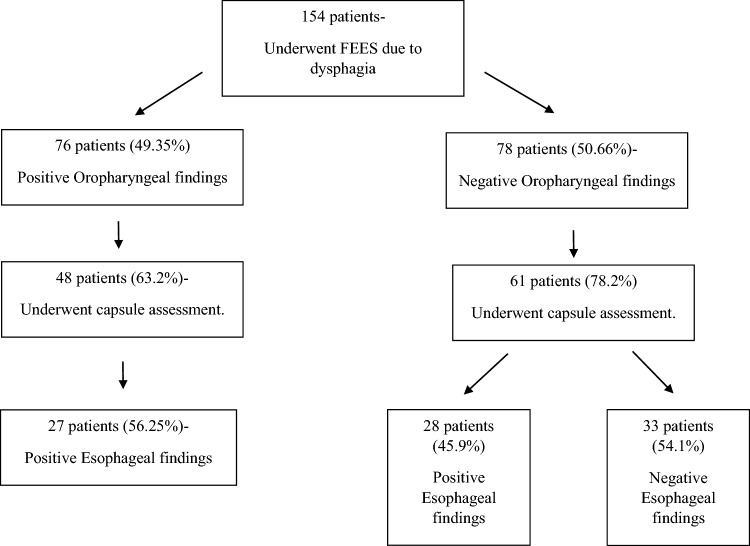
Flow chart of the distribution of the patients in the study

Based on the standard FEES exam, 76 patients (49.35%) were diagnosed with oral, pharyngeal, or oropharyngeal swallowing disorders, and 78 patients (50.64%) had a normal standard FEES exam. The distribution of the patients based on their test results are shown in Fig. 2.

The empty capsule test was not performed in 45 patients (29.2%), as shown in Fig. 2. Of them, nine (23.68%) refused to proceed with the capsule test following the completion of the standard FEES exam. Fourteen patients (36.8%) were found to have severe oropharyngeal dysphagia by the standard FEES exam, and the medical team decided to avoid the capsule test.

Although the standard FEES exam was normal in 15 patients (39.47%), the medical team decided not to proceed with the capsule test due to severe comorbidities or high sensitivity to the endoscope in the lower pharynx. The test was well tolerated among all patients in which the endoscope was inserted into the esophagus, with only mild discomfort.

The distribution of modified FEES results are shown in Table 3. Sixty-three (57.7%) patients demonstrated pathological capsule swallowing in either the oral, pharyngeal, or esophageal phases. Of them, 55 patients (87.3%) showed esophageal swallowing pathology, 28 of which were isolated esophageal dysphagia, and 27 had combined dysphagia (Fig. 2). In Five patients (7.9%), the capsule test showed pharyngeal dysphagia, two of them combined with esophageal dysphagia. All five patients had already been diagnosed using the standard FEES exam. An additional six patients had oral dysphagia, one of them combined with esophageal dysphagia. Of those six patients, three were diagnosed by capsule swallowing only.

**Table 3:** FEES examination results

	Total	Capsule positive (n = 55)	Capsule negative (n = 54)	p Value
IDDSI 0				0.609
Normal	78 (71.6%)	39 (70.9%)	39 (72.2%)	
Oral dysphagia	1 (0.9%)	1 (1.8%)	0 (0%)	
Pharyngeal dyspagia	30 (27.5%)	15 (27.3%)	15 (27.8%)	
IDDSI 3				0.813
Normal	6 (5.5%)	2 (3.6%)	4 (7.4%)	
Oral dysphagia	0 (0%)	0 (0%)	0 (0%)	
Pharyngeal dysphagia	3 (2.8%)	2 (3.6%)	1 (1.9%)	
Wasn’t conducted	100 (91.7%)	51 (92.7%)	49 (90.7%)	
IDDSI 4				0.582
Normal	84 (77.1%)	43 (78.2%)	41 (75.9%)	
Oral dysphagia	1 (0.9%)	1 (1.8%)	0 (0%)	
Pharyngeal dysphagia	14 (12.8%)	8 (14.5%)	6 (11.1%)	
Wasn’t conducted	10 (9.2%)	3 (5.5%)	7 (13.0%)	
IDDSI 5 traditional				0.848
Normal	11 (10.1%)	4 (7.3%)	7 (13.0%)	
Oral dysphagia	0 (0%)	0 (0%)	0 (0%)	
Pharyngeal dysphagia	2 (1.8%)	0 (0%)	2 (3.7%)	
Wasn’t conducted	96 (88.1%)	51 (92.7%)	45 (83.3%)	
IDDSI 6				0.157
Normal	2 (1.8%)	2 (3.6%)	0 (0%)	
Oral dysphagia	0 (0%)	0 (0%)	0 (0%)	
Pharyngeal dysphagia	0 (0%)	0 (0%)	0 (0%)	
Wasn’t conducted	107 (98.2%)	53 (96.4%)	54 (100%)	
IDDSI 7				0.233
Normal	73 (67.0%)	34 (61.8%)	39 (72.2%)	
Oral dysphagia	0 (0%)	0 (0%)	0 (0%)	
Pharyngeal dysphagia	29 (26.6%)	18 (32.7%)	11 (20.4%)	
Wasn’t conducted	7 (6.4%)	3 (5.5%)	4 (7.4%)	
IDDSI 7 traditional				0.496
Normal	22 (20.2%)	13 (23.6%)	9 (16.7%)	
Oral dysphagia	1 (0.9%)	0 (0%)	1 (1.9%)	
Pharyngeal dysphagia	10 (9.2%)	6 (10.9%)	4 (7.4%)	
Wasn’t conducted	76 (69.7%)	36 (65.5%)	40 (74.1%)	
IDDSI 7 EC traditional				0.267
Normal	16 (14.7%)	8 (14.5%)	8 (14.8%)	
Oral dysphagia	1 (0.9%)	0 (0%)	1 (1.9%)	
Pharyngeal dysphagia	5 (4.6%)	4 (7.3%)	1 (1.9%)	
Wasn’t conducted	87 (79.8%)	43 (78.2%)	44 (81.5%)	

Following modified FEES results, patients were advised to proceed with gastroenterology evaluation (GE). Of them, 42 (75%) had completed the GE (Table 4). In 26 patients (61.9%; 26/42), an esophageal disorder such as gastroesophageal reflux, hiatal hernia, eosinophilic esophagitis, esophageal web, or motility disorder was diagnosed by one of the tests. Gastroesophageal pathologies in patients who underwent capsule examination are described in Table 5.

**Table 4:** correlation with gastroenterological evaluation

	Total (n = 109)	Capsule positive (n = 55)	Capsule negative (n = 54)	p Value
Gastroscopy				< 0.001
Normal	20 (18.3%)	7 (12.7%)	13 (24.1%)	
Esophageal dysphagia	21 (19.3%)	19 (34.5%)	2 (3.7%)	
Wasn’t conducted	68 (62.4%)	29 (52.7%)	39 (72.2%)	
Manometry				0.0607
Normal	8 (7.3%)	2 (3.6%)	6 (11.1%)	
Esophageal dysphagia	10 (9.2%)	8 (14.5%)	2 (3.7%)	
Wasn’t conducted	91 (83.5%)	45 (81.8%)	46 (85.2%)	
Fluoroscopy				0.0118
Normal	5 (4.6%)	1 (1.8%)	4 (7.4%)	
Esophageal dysphagia	7 (6.4%)	7 (12.7%)	0 (0%)	
Wasn’t conducted	97 (89.0%)	47 (85.5%)	50 (92.6%)	
Total gastroenterological exams				< 0.001
Normal	16 (14.7%)	4 (7.3%)	12 (22.2%)	
Esophageal dysphagia	26 (23.9%)	23 (41.8%)	3 (5.6%)	
Wasn’t conducted	67 (61.5%)	28 (50.9%)	39 (72.2%)

**Table 5:** Gastroesophageal pathologies in patients who underwent Capsule examination

Patients number	Age	significant comorbidities	FEES diagnosis	Capsule diagnosis	GI diagnosis
1	57	Diabetes Mellitus	Normal	Esophageal hypomobility	Esophageal web, Hiatal Hernia
2	81	Diabetes Mellitus	Pharyngeal dysphagia	Capsule seen in the esophagus	Tertiary esophageal contractions, esophageal reflux
3	79	No comorbidities	Normal	No peristalsis, Capsule seen in the esophagus	Ineffective peristalsis, Hiatal Hernia
4	78	Diabetes Mellitus	Normal	Upward peristalsis, Capsule seen in the esophagus	High PH
5	86	No comorbidities	Normal	No peristalsis, Capsule seen in the esophagus	Esophageal reflux, Gastritis
6	78	No comorbidities	Normal	Capsule seen in the esophagus	Gastritis, High pressure in lower esophageal sphincter
7	65	Cerebrovascular accident	Normal	Capsule seen in the esophagus	Esophagitis, esophageal reflux
8	69	Vocal cord paresis	Pharyngeal dysphagia	Capsule seen in the esophagus	Hiatal Hernia
9	69	Diabetes Mellitus, Cerebrovascular accident	Normal	Normal	High pressure in upper esophageal sphincter
10	80	Parkinson’s disease	Pharyngeal dysphagia	Normal	Esophagitis, esophageal reflux
11	71	Diabetes Mellitus	Pharyngeal dysphagia	No peristalsis	Hiatal Hernia
12	69	No comorbidities	Normal	Capsule seen in the esophagus	Hiatal Hernia
13	76	No comorbidities	Normal	Capsule seen in the esophagus	Hiatal Hernia
14	79	Diabetes Mellitus	Pharyngeal dysphagia	No peristalsis	High pressure in midesophagus
15	74	No comorbidities	Normal	Normal	Hiatal Hernia
16	47	No comorbidities	Normal	Capsule seen in the esophagus	Ineffective esophageal motility, Hiatal Hernia
17	85	No comorbidities	Pharyngeal dysphagia	Capsule seen in the esophagus	Esophageal candidiasis,
18	83	No comorbidities	Pharyngeal dysphagia	Normal	Hiatal Hernia
19	71	Diabetes Mellitus, Cerebrovascular accident	Pharyngeal dysphagia	Capsule seen in the esophagus	Hiatal Hernia
20	75	Diabetes Mellitus	Normal	Capsule seen in the esophagus	Esophageal diverticula, High pressure in lower esophageal sphincter
21	77	Cerebrovascular accident	Normal	Normal	Esophagitis
22	75	Diabetes Mellitus	Normal	Capsule seen in the esophagus	Hiatal Hernia
23	70	Parkinson’s disease	Pharyngeal dysphagia	No peristalsis	Ineffective esophageal motility
24	74	No comorbidities	Pharyngeal dysphagia	No peristalsis	Hiatal Hernia
25	48	No comorbidities	Normal	Normal	Ineffective esophageal motility

Among the 27 patients with a pathological capsule test, the diagnosis of 23 patients (85.1%) was verified by following GE. Among the 15 patients who had a normal capsule test and were recommended GE, three patients (20%) were found to have an esophageal pathology (Table 4).

Predictive values of modified FEES compared with GE tests are presented in Table 6. Compared with gastroscopy (n = 20), capsule swallowing for esophageal diagnosis had an accuracy of 78%, sensitivity of 90%, specificity of 65%, PPV of 73% and NPV of 86.6%. Compared with manometry (n = 9), capsule swallowing demonstrated a 78% accuracy, 80% sensitivity, 75%specificity, 80% PPV and 75% NPV. Compared with fluoroscopy (n = 7), capsule swallowing showed an accuracy of 91.67%, sensitivity of 100%, specificity of 80%, PPV of 87.5% and NPV of 100%. Compared with all gastroenterological exams, capsule swallowing demonstrated an 83.3% accuracy, 88.5% sensitivity, 75%specificity, 85% PPV, and 80% NPV (Table 6).

**Table 6:** Accuracy tests and AUC

	Total accuracy	Sensitivity	Specificity	PPV	NPV	AUC
Gastroscopy	78%	90%	65%	73%	86.6%	0.777
Manometry	78%	80%	75%	80%	75%	0.775
Fluoroscopy	91.67%	100%	80%	87.5%	100%	0.900
Total gastroenterological exams	83.33%	88.46%	75%	85.18%	80%	0.817

The area under the curve was 0.777 for gastroscopy, 0.775 for manometry, 0.900 for fluoroscopy, and 0.817 for total gastroenterological exams (Table 5).

## Discussion

While the FEES exam is not designed to diagnose esophageal causes for dysphagia, its advantages as an affordable and accessible test have led the authors to create a modification that may be utilized for an initial assessment of esophageal causes of dysphagia. The purpose of this pilot study was to evaluate the clinical benefit of a modification to the commonly used FEES examination by adding a capsule swallowing assessment combined with a proximal flexible esophagoscopy using standard nasoendoscopy. The modified FEES exam demonstrated high predictive values of esophageal dysphagia when compared to additional tests taken, suggesting its potential role as a screening tool. To the best of our knowledge, this is the first study to suggest FEES as an alternative for an initial esophageal diagnosis using the capsule modification.

A comprehensive assessment of the causes of esophageal dysphagia may require multiple tests, including esophagoscopy, barium esophagography, and high-resolution esophageal manometry, each with its advantages and diagnostic ability of different esophageal pathology [5, 10–13]. The multiple tests required and their nature (sedation for EGD, inconvenience of esophageal manometry, and radiation exposure of MBS) are both time-consuming and costly.

In this research, only 48.2% of the patients were adherent to the medical recommendations for further GE evaluation. This finding stresses the importance of providing a simple, available tool for esophageal dysphagia assessment within the FEES exam, further verifying the clinical role of modified FEES as an alternative. The adherence rate for GE following FEES has not been investigated to date, which warrants its evaluation in future studies.

An important potential benefit of the modified FEES exam, which was not quantified and evaluated in this study, is its tolerability. GE tests were reported to have low tolerability, especially among patients with severe comorbidities [14]. On the other hand, the safety and tolerability of the FEES exam are well established in previous studies [15, 16]. Trans nasal esophagoscopy tolerability has also been evaluated in previous studies [17, 18]. In a series of 50 patients, Sharma et al. demonstrated that the majority of patients scored very low on the visual analog scale [19]. The mean and median pain scores were 1. The discomfort included nasal pain, throat pain, and nausea. Streckfuss et al. [20] used a visual analog scale of 1–5 and also found a mean result of ≤ 2. They also evaluated which endoscope position caused the most discomfort to the patient. It was found that the endoscopy of the pharynx seemed to cause the most discomfort, while the passage of the nose and esophagus appeared to be perceived as less unpleasant. In this current study, the selected patients tolerated the capsule test well. Yet, the authors suggest that the evaluation of the upper esophagus during the FEES exam should be avoided in patients with highly sensitive gag reflex, patients with known upper esophagus disorder such as Zenker’s diverticulum, or in patients with severe comorbidities and should be further assessed in future studies.

Limitations of this study include its small sample size and the willingness of participants to consent to the study. Only a few of the patients underwent FEES with capsule swallowing, and an even smaller subset underwent further gastrointestinal evaluation. In addition, this study does not have a control group, which may impact the conclusion. Moreover, patients’ satisfaction and tolerability of the exams were not directly assessed in the study. Due to the small amount of gastrointestinal evaluation exams, it remains unclear what kind of exam should be recommended for those patients.

## Conclusions

Capsule swallowing as a part of the FEES examination may be useful for initial esophageal assessment and should be considered as an additional test for the standard FEES exam. Further studies are required to validate its clinical benefit, as well as patients’ satisfaction and tolerance.

## Data Availability

All data supporting the findings of this study are available within the paper and its Supplementary Information.
